# Effect of Adding a Six-Week Course of Doxycycline to Intensive Hygiene-Based Care for Improving Lymphedema in a Rural Setting of Mali: A Double-Blind, Randomized Controlled 24-Month Trial

**DOI:** 10.4269/ajtmh.23-0908

**Published:** 2024-07-16

**Authors:** Yaya I. Coulibaly, Abdoul F. Diabate, Moussa Sangare, Sekou O. Thera, Housseini Dolo, Salif S. Doumbia, Siaka Y. Coulibaly, Ayouba Diarra, Lamine Diarra, Diadje Tanapo, Michel E. Coulibaly, Lamine Soumaoro, Abdallah A. Diallo, Amatigue Zeguime, Yacouba Sanogo, Adama Berthe, Fatoumata Dite Nene Konipo, Charles Mackenzie, Mariana Stephens, Joseph P. Shott, Jayla Norman, Ute Klarmann-Schulz, Achim Hoerauf, Andrew Majewski, John Horton, Sarah Sullivan, Eric A. Ottesen, Thomas B. Nutman

**Affiliations:** ^1^ International Center for Excellence in Research, Bamako, Mali;; ^2^ Dermatology Hospital of Bamako, Bamako, Mali;; ^3^ Neglected Tropical Diseases Support Center, Task Force for Global Health, Decatur, Georgia;; ^4^ The Reaching the Last Mile Fund, The End Fund, New York, New York;; ^5^ Division of Neglected Tropical Diseases, Global Health Bureau, Bethesda, Maryland;; ^6^ Institute for Medical Microbiology, Immunology and Parasitology, German Centre for Infection Research (DZIF), Bonn-Cologne Site, University Hospital Bonn, Bonn, Germany;; ^7^ Tropical Projects, Hitchin, United Kingdom;; ^8^ National Institute of Allergy and Infectious Diseases, Bethesda, Maryland

## Abstract

Lymphedema (LE) is one the most disfiguring chronic manifestations of lymphatic filariasis. Its management relies primarily on limb hygiene and local care. A previous study in Ghana demonstrating a beneficial effect of doxycycline on LE led to the current multicenter trial on the efficacy of doxycycline in filarial LE. A randomized placebo-controlled trial was initiated in two rural health districts in Mali. Patients with LE stages 1–3 were randomized to receive either doxycycline (200 mg/day) or placebo over a 6-week monitored treatment period and were then followed every 6 months for 2 years. Both groups received materials for limb hygiene that was carried out daily for the entire 2-year study. The primary endpoint was lack of progression in LE stage at 24 months. One hundred patients were enrolled in each study arm. The baseline sociodemographic characteristics of each group were largely similar. There was no significant difference at month 24 after treatment initiation in the number of subjects showing progression in LE stage between the two treatment arms (*P* = 0.5921). Importantly, however, the number of attacks of acute adenolymphangitis (ADLA) was reduced in both arms, but there was no significant difference between the two groups at any follow-up time point (all *P* >0.23). Doxycycline was well tolerated in those receiving the drug. When added to daily self-administered limb hygiene, a 6-week course of doxycycline (200 mg) was not superior to placebo in increasing the improvement associated with hygiene alone in LE volume, stage, or frequency of ADLA attacks over a 24-month period.

## INTRODUCTION

Lymphatic filariasis (LF), a neglected tropical disease, is caused by an infection with the filarial nematodes *Wuchereria bancrofti*, *Brugia malayi*, or *Brugia timori*. The main clinical manifestations of LF are hydrocele and lymphedema (LE),^1^ both of which (when severe) are associated with stigma, diminished quality of life, and economic loss.^2^^,^^3^ Based on this burden of disease, the WHO and its partners targeted LF for elimination as a public health problem by 2030.^4^^,^^5^

The global strategy for LF elimination relies on two main pillars: 1) transmission interruption using annual mass drug administration (MDA) and 2) morbidity management and disability prevention to alleviate suffering.^6^^,^^7^ Although significant progress in LF elimination has been made in transmission interruption across the world,^8^ including most evaluation units in Mali,^9^ relatively little progress has been made in the area of morbidity control. Morbidity control is based largely on surgery for hydrocele and a basic package of hygiene care for LE. Prior to the launch of the Global Program to Eliminate Lymphatic Filariasis (GPELF) in 1997, among those 130 million with LF infection, there were estimated to be 30 million patients with hydrocele and 18 million with LE.^10^ By 2014, data indicated that MDA had prevented 97 million cases, representing a 59% reduction in global LF burden.^10^ However, this improvement was largely related to a reduction of those with microfilaremia through the widespread adoption of MDA. For morbidity management, less dramatic changes were observed based largely on the failure of national programs to provide resources needed to ameliorate/stabilize LE and hydrocele.^11^ As of 2013, only 27 of the original 83 countries where LF is endemic reported morbidity management and disability prevention intervention programs. According to the first estimates of LF worldwide, 25 million men suffered from hydrocele and over 15 million people from LE.^8^ Thus, even if the goal of interruption of transmission is achieved globally and infection in subsequent generations is no longer an issue, those with LE and hydrocele face a lifetime of progressive disability.

Treatment of LE relies largely on the reduction in frequency and severity of acute adenolymphangitis (ADLA). The mainstay of care is hygiene (daily washing with soap and water) of affected limbs with the added use of fungicidal/bactericidal topical cream and exercise and limb elevation intended to improve lymphatic flow.^12^ This “hygiene” package can stabilize or modestly reverse the evolution of LE pathogenesis but requires the patient to adhere rigorously to a daily regimen that may be difficult to achieve in rural areas of many low- to middle-income countries where education levels are low and poverty prevents acquisition of the materials needed for correct use of this package of care.

Doxycycline has been shown to be an effective therapy to kill or render infertile *Wuchereria* and *Brugia* adult worms through its effect on their *Wolbachia* endosymbiont.^13^^–^^15^ Additionally, a 6-week course of doxycycline once daily was reported as potentially effective at preventing LE progression in *W. bancrofti-*infected patients in Ghana.^14^^,^^16^ It was also shown to decrease the severity of mild to moderate LE (stages 1–3) in those without active filarial infection (e.g., circulating antigen-negative individuals).^14^ These data implied that the efficacy of doxycycline in LE improvement might be unrelated to its anti-*Wolbachia* effects.^14^

To corroborate these findings, a multicenter, multicountry study to assess the impact of doxycycline (in addition to standard self-administered “hygiene” treatment) on LE was initiated in six countries (Ghana, Tanzania, Mali, Sri Lanka, and India [LF] as well as Cameroon [to also include podoconiosis]) using common protocols and endpoints.^17^ The present study describes the effect of doxycycline (plus hygiene) and its suitability for use in remote/rural areas of LF endemicity in Mali, where most LE cases are found.^18^

## MATERIALS AND METHODS

### Design and protocol.

**Figure 1.**
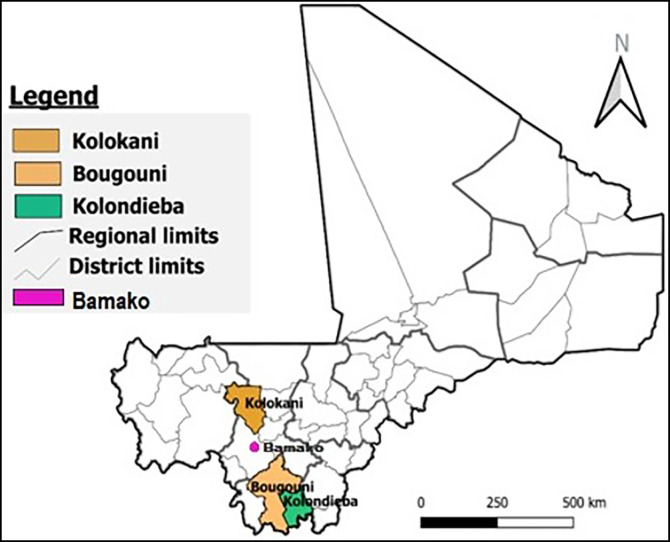
Map of Mali showing the study sites.

This study (LEDoxy) was designed as a multicenter, randomized, controlled, double-blind superiority study with the primary endpoint being a LE stage change at 24 months. The study in Mali was conducted in populations of patients (Table 1) having LE (stages 1–3) based on the Dreyer scale.^19^ The decision/criteria to enroll only patients with LE stages 1–3 was based on previous studies’ conclusions. Mand et al.^14^ explicitly recommended a 6-week course of doxycycline as an improved tool to manage LE in patients with stages 1–3. Patients with LE in Mali are generally found in rural areas, with one to two cases in a village. Based on a comprehensive survey of LE in three health districts of Mali (Kolondieba, Bougouni, and Kolokani) (Figure 1),^18^ all those previously identified as having LE were screened for eligibility. Those subjects, after providing written consent, were assigned to a treatment arm using block randomization as previously described.^17^

**Table 1:** Baseline characteristics of the study population

Variable	Doxycycline Group	Placebo Group	Total	*P*-Value*
Number of Patients (*N*)	100	100	200	–
Gender (*n*)				
Male	13	13	26	1
Female	87	87	174	
Weight Group (*n*)				
≤50 kg	16	9	25	0.19
>50 kg	84	91	175	
Age (years)				
Mean ± SD	52.5 ± 10.5	52.7 ± 9.64	52.6 ± 10.1	0.79
95% CI	51.60–53.40	51.90–53.60	52.00–53.20	
Duration of Residence in Area of LF Endemicity (years)				
Mean ± SD	47.8 ± 11.8	47.5 ± 13.0	47.7 ± 12.4	0.97
95% CI	48.8–48.9	46.3–48.6	46.9–48.4	
History of Participation in MDA (*n*)				
Yes	39	37	76	0.75
No	40	43	83	
Unknown	21	20	41	
Number of ADLA Attacks in Past Year				
Mean ± SD	1.44 ± 4.32	1.10 ± 1.23	1.26 ± 3.13	0.16
Range	1.03–1.85	0.986–1.21	1.06–1.47	
Duration of Last ADLA Attack (days)				
Mean ± SD	9.83 ± 0.98	7.31 ± 0.54	–	0.23
95% CI	8.62–1.00	6.52–8.10	–	
FTS Result (*n*)				
Positive	9	12	21	0.65
Negative	91	88	179	

ADLA = acute adenolymphangitis; FTS = filariasis test strip; LF = lymphatic filariasis; MDA = mass drug administration; *N* = number.

* *P*-value comparing differences between doxycycline and placebo treatment groups.

### Study sites.

The study was carried out in the health districts of Kolondieba of the Sikasso region and Kolokani of the Koulikoro region (Figure 1). These two health districts, according to our preliminary investigations with health care providers and the National Program To Eliminate Lymphatic Filariasis of Mali, are the health districts where a high number of carriers of chronic clinical signs of LF live. Villages geographically close to neighboring health districts were included if more than one case was found in a village or hamlet. This was the case of the district of Bougouni.

The Kolondieba health district covers an area of 920 km2, with a total population estimated at around 216,260 inhabitants in 2017 based on the national census of 2009,^20^ living in a total of 205 villages. This health district is in the center of the Sikasso region and is also the fourth largest district of the region. It is bordered to the north by the Bougouni health district, to the south by the Republic of Côte d’Ivoire, to the east by the Sikasso health district, and to the west by the Kadiolo health district. The Kolondieba health district is located 240 km from Bamako, the capital city. Kolondieba has a tropical climate with an average annual rainfall of 1,250 mm. Subsistence farming is the main occupation, along with gold mining and forest exploitation.

The Kolokani health district covers an area of 14,380 km2, with a total population estimated at 287,380 inhabitants in 2017 based on the national census of 2009.^20^ This population is spread across 50 villages. This health district is in the Koulikoro region. It is bordered to the northeast by the Nara health district, to the south by the Kati health district, to the east by the Koulikoro health district, and to the west by the Diéma health district. The Kolokani health district is located 105 km from Bamako, the capital city. Kolokani has a tropical climate, with an average annual rainfall of 589.4 mm. Subsistence farming is the main occupation, along with forest exploitation.

### Sample size.

The study sample size was calculated using the findings from a previous study,^14^ noting that progression of LE occurred in 55% of those given placebo compared with 5% given doxycycline. Based on this, the minimal required sample size was 70 patients per treatment arm to detect a statistical difference between the two treatment arms with a significance level of 5% and a 95% power. We considered the possibility of a 30% rate of subject loss over the 2 years, thereby requiring a sample size of 100 patients per treatment arm.

### Intervention.

Eligible patients were randomized to receive either doxycycline or placebo daily for 6 weeks (42 days). Doxycycline hyclate 100-mg tablets (Remycin™; Remedica, Limassol, Cyprus) were used with a matching placebo produced by Piramal Healthcare, Morpeth, United Kingdom. Packaging the treatment packs for the study was performed by Piramal Healthcare, Morpeth, United Kingdom.^17^ Doxycycline and placebo were administered under supervision (directly observed treatment) for 6 weeks. The first dose of doxycycline (two 100-mg tablets for those weighing more than 50 kg or one 100-mg tablet for those weighing between 40 and 50 kg) or placebo was administered after all screening assessments, administration of informed consent or assent, and patient integration into the basic hygiene program. Subjects were asked to have eaten prior to swallowing the tablets with 120 mL of water. Vomited doses were replaced.

### Hygiene.

All subjects participated in a hygiene program for limb care based on the principles established in the New Hope booklet for LE patients.^19^ A generic standard operating procedure (SOP) was used with cards in French that described how to use the hygiene methods so that there was uniformity of training and use across investigators. Subjects were trained during contact at all stages of the study (baseline and months 3, 6, 12, and 18). All the training sessions and questionnaire administration with study participants were done using local languages.

### The WHO Disability Assessment Schedule (WHODAS).

Quality of life was assessed at baseline and at months 12 and 24 after treatment initiation using the WHODAS 2.0 tool, a generic health disability assessment tool grounded in the conceptual framework of the International Classification of Functioning, Disability and Health. It captures an individual’s level of function in six major life domains, half related to physical disabilities and half to nonphysical disabilities. This version of the 12-item self-reporting WHODAS 2.0 tool had previously been translated and validated in the local language.^21^

### Treatment assessment.

At baseline (day 0), a complete clinical history, physical examination, and laboratory assessment (complete blood count, liver function tests, creatinine, blood urea nitrogen) were performed. Assessment of LE (see below), its clinical appearance using photographs, and the presence of acute attacks were documented. Patients received drug supplies weekly and were supervised by a family member or another village inhabitant termed the “person in charge.” A local drug distributor or health professional was selected for this role when available. Regular phone calls allowed close follow-up and management of adverse events. The medical officer from the nearest health center to the patients was trained as the medically responsible person to monitor and assess adverse events in real time.

**Figure 2.**
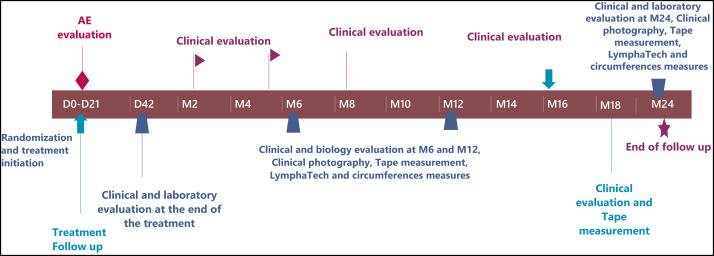
Study implementation design showing the main time points. D = day; M = month.

At major study time points, patients were invited to the health center nearest to their village for an assessment in line with the standardized protocol for all subjects (Figure 2). Unannounced visits at participants’ houses were conducted by study physicians to ascertain the number of remaining tablets and the effectiveness of limb hygiene.

### Lymphedema assessment (baseline and at months 6, 12, 18, and 24).

Three techniques were used to gauge the LE at baseline and each assessment.

First, the circumference of each leg was measured with a tape measure at 10 cm from the tip of the big toe and at 12, 20, and 30 cm from the sole of the foot. The average of each of the four measurements was calculated before treatment and at each follow-up visit.

Second, ultrasound of the legs to assess LE was performed after standardized training for competency as described by Mand et al.^14^ Patients were examined between 2:00 pm and 7:00 pm. For each patient, repeat scans were performed within a 2-hour (±1 hour) window at each visit to ensure consistency throughout the trial, using a portable ultrasound device equipped with a 38-mm, 5- to 10-MHz linear-position transducer (Lumify probe; Philips, Andover, MA). Ultrasound was performed on patients sitting with legs extended and feet perpendicular to the legs. The transducer was positioned over the malleolus at a 90° angle to the cross-sectional skin surface. The thickness of the malleolar tissue (hypodermis, dermis, and epidermis) was measured at the skin surface. Skin thickness over the lateral and medial malleoli of both legs was measured before treatment and at follow-up.

Third, a LymphaTech^®^ scanner tool was used to measure volume differences using infrared light emission scanning technology. Use of the scanner followed extensive training and SOPs. Scanning was performed immediately before or after the ultrasound, at about the same time of day for each individual at every evaluation (±2 hours) and in the same position to get reproducible and comparable results. The validation information and utility of the LymphaTech scanner has been described previously.^22^

### Laboratory evaluation.

At baseline and at day 21, day 42, month 6, month 12, and month 24, venipuncture was performed to assess the profile of hematologic, hepatic, and renal parameters; and at baseline, circulating filarial antigen was assessed using a filariasis test strip (Alere, Scarborough, ME). Urine pregnancy tests were performed at baseline and at 3 and 8 weeks after the start of the 6-week-long course of doxycycline/placebo for women of childbearing age. Biological samples were transported in an air-conditioned vehicle to Bamako and processed within 6 hours. Biochemical (creatinine, aspartate aminotransferase, alanine aminotransferase, and gamma-glutamyl transferase) and hematological (blood cell count) analyses were carried out by the College of American Pathologists (CAP)-accredited clinical laboratory at the International Center for Excellence in Research in Mali (ICER-Mali), which works closely with the NIH. The remaining blood samples were frozen immediately and sent to the laboratory of the Neglected Tropical Diseases Research Unit of the ICER-Mali.

### Case report forms, monitoring, and data entry/database.

Research data were recorded in paper format on case report forms before being double entered into the Research Electronic Data Capture (REDCap) tools hosted at Emory University.^23^^,^^24^ REDCap is a secure, Health Insurance Portability and Accountability Act (HIPAA)-compliant, Web-based software platform designed to support data capture for research studies, providing 1) an intuitive interface for validated data capture, 2) audit trails for tracking data manipulation and export procedures, 3) automated export procedures for seamless data downloads to common statistical packages, and 4) procedures for data integration and interoperability with external sources. We used a mobile application for tablets equipped with an iOS operating system for data capture. All electronic devices were password protected. Digital data captured using the LymphaTech scanner were collected and uploaded securely along with the participant’s identification number, reference number, and date of examination to a separate secure server. All clinical and laboratory procedures were performed according to standard protocols governed by good clinical practice (GCP) and good laboratory practice guidelines. A single independent data safety monitoring board of four medical experts was established to support the studies in Mali, Sri Lanka, and India. They received quarterly reports of adverse events from each site, met by phone with the study’s safety officer to assess all reports of adverse events every 6 months, and considered individual serious adverse events immediately whenever they occurred.

Independent study monitoring was conducted onsite for data and process integrity by a clinical research organization (FHI Clinical, Durham, NC) at study initiation, during the treatment phase at 12 months, and virtually, because of COVID-19 restrictions, at the end of the study. Additionally, an audit to ensure that everything in the study and data collection was GCP compliant was performed by an independent auditor arranged by the clinical research organization at month 6 after study initiation.

## STATISTICAL ANALYSES

Descriptive statistics were calculated for all baseline variables, and bivariate analyses were conducted to determine if randomization yielded treatment and control groups with similar characteristics at baseline. Frequencies were calculated for categorical variables, and Fisher’s exact tests were used. For continuous variables, descriptive statistics were calculated (mean, standard deviation, minimum, maximum, range), and *t* tests were performed to assess differences between doxycycline and placebo. For continuous variables that were nonnormal, nonparametric statistics were used.

Outcomes at 6, 12, and 24 months were assessed for differences between the doxycycline and placebo groups while both groups were concomitantly receiving the standardized hygiene practice training and support. Differences between doxycycline and placebo groups were compared using two-sided hypotheses with an α of 0.05 and 95% CIs. Categorical variables were analyzed using Fisher’s exact tests. Ordinal variables, such as whether LE stage progressed, stayed the same, or improved, were assessed using the Jonckheere-Terpstra test for trend. Kaplan-Meier curves were used to visualize time to first ADLA attack, and median “survival times” were calculated.

Mixed effects models with a time effect were used to determine whether differences in the measurement endpoints exist between study groups over time. Study group and time were the main effects. Baseline characteristics, e.g., sex, age, and disease history, were included as covariates. Survival analysis techniques were used to assess whether differences in time to an acute attack exist between the placebo and treatment groups. Cox proportional hazards regression was used to assess the impact of the treatment adjusted for other covariates.

All analyses were conducted on the intention-to-treat population. Statistical analyses were performed using SAS 9.4 (SAS Institute, Cary, NC) and GraphPad Prism (v. 10.0.0; GraphPad Software, San Diego, CA).

## RESULTS

### Participants.

**Figure 3.**
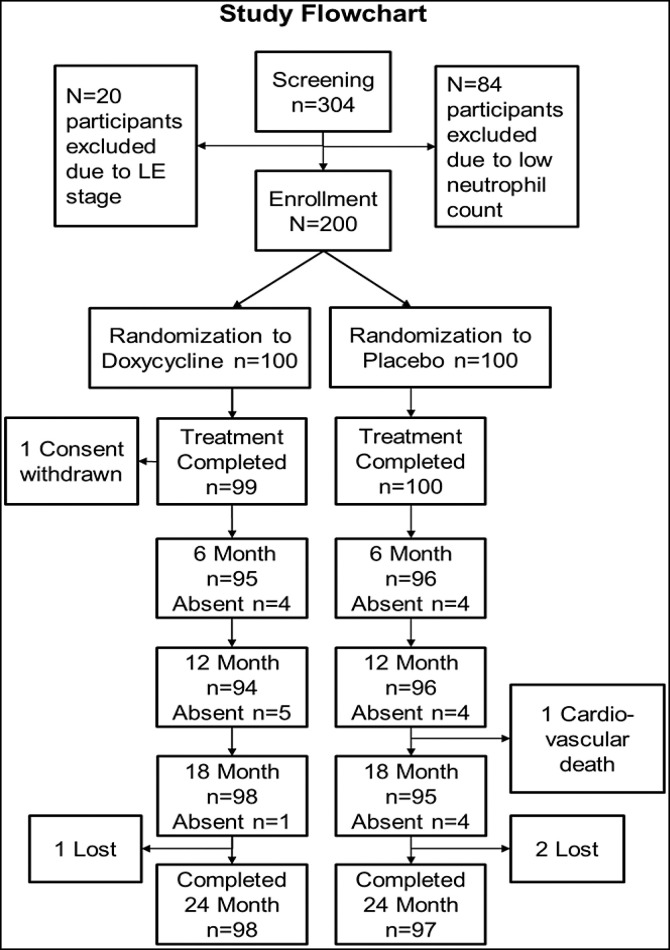
Study flow chart. LE = lymphedema.

Of 304 patients with LE identified in the three health districts (see map in Figure 1), 200 were enrolled in the current study. As can be seen in Figure 3, 20 of 304 participants screened were excluded based on the strict LE staging criteria and 84 of 304 were excluded because of low absolute neutrophil counts. Low neutrophil counts are frequently encountered in adults in sub-Saharan Africa without sequelae,^25^ but these patients were excluded to ensure more homogeneous study populations across all trial sites. The 200 enrolled patients received the 6-week treatment course with either doxycycline or placebo: one subject (placebo group) retracted consent after 4 days of treatment. A few patients were absent at the intermediate follow-up visits, but all available subjects were present for the last visit at 24 months after treatment initiation. Overall, only three patients in the doxycycline group and two patients in the placebo group were lost at the 2-year follow-up time point (Figure 3).

### Baseline data.

The study participants in the two treatment arms (*N* = 100 in each) were largely similar, as shown in Table 1. There was no difference between the doxycycline and placebo treatment arms for any of the parameters assessed. These included sex, age range, weight, mean duration of exposure, previous participation in MDA, the number of ADLA attacks over the previous 12 months, or the proportion of participants with antigenemia (based on filariasis test strip). Lymphedema stage frequencies, LE volumes using LymphaTech (in mL), skin thicknesses using ultrasound, and LE circumferences (in cm) using a tape measure (Table 2) were comparable between the two treatment arms.

**Table 2:** Baseline LE assessment

Variable	Doxycycline Group	Placebo Group	Total	*P*-Value
LE Stage Number				
1	11	6	17	0.41
2	44	43	87	
3	45	51	96	
LymphaTech Volume (mL)	Median = 2,528; range, 1,520–4,200	Median = 2,668; range, 1,755–4,410	Median = 2,590; range, 1,520–4,410	0.028
Ultrasound				
Medial Malleolus (cm)	Median = 0.61; range, 0.18–1.59	Median = 0.71; range, 0.17–2.59	Median = 0.66; range, 0.17–2.59	0.0458
Lateral Malleolus (cm)	Median = 0.4; range, 0.11–1.44	Median = 0.43; range, 0.14–1.69	Median = 0.44; range, 0.11–1.69	0.104
Circumference Measured at Specified Points of Leg (cm)*				
10	23.7 (19.6–31.3)	24.1 (20.0–31.5)	24.0 (19.6–31.5)	0.078
12	24.4 (17.9–34.3)	25.3 (19.1–38.9)	24.9 (17.9–38.9)	0.178
20	27.9 (19.3–37.9)	29.0 (20.4–42.3)	28.3 (19.3–42.3)	0.075
30	33.7 (25.2–42.9)	34.4 (24.9–49.8)	34.0 (24.9–49.8)	0.118

LE = lymphedema.

* Circumferences were measured at specified points of the affected leg (see Materials and Methods). Data presented as median (range).

### Primary endpoint analysis.

With the primary endpoint of the study being progression to a higher LE stage at 24 months (Table 3), we could show that there were no differences at any time point in the number of subjects showing worsening of LE stage between the two treatment arms. At 24 months, only 6/98 (6%) subjects in the doxycycline arm and 9/97 (9%) in the placebo arm showed progression to more severe LE stages. Thus, the primary endpoint set for defining the potential efficacy of doxycycline (beyond hygiene) in filarial LE did not discriminate between the doxycycline and placebo groups.

**Table 3:** LE grade change over the 24-month study period by treatment arm

Treatment Arm and Change*	Patients [*n* (%)] with LE Grade Change in Indicated Follow-up Period (posttreatment)		
	M6, *n* (%)	M12, *n* (%)	M24, *n* (%)
Doxycycline			
Improvement	25 (26)	35 (37)	40 (41)
No Change	65 (68)	54 (57)	52 (54)
Worsening	5 (5)	5 (5)	6 (6)
Placebo			
Improvement	28 (29)	29 (30)	36 (37)
No Change	63 (66)	58 (60)	52 (54)
Worsening	5 (5)	9 (9)	9 (9)
*P*-value	0.7213	0.2193	0.4927

LE = lymphedema; M6 = visit at 6 months; M12 = visit at 12 months; M24 = visit at 24 months; *n* = number.

* Improvement = decrease in LE grade; no change = no change in LE grade; worsening = increase in LE grade.

### Secondary outcome analyses.

**Figure 4.**
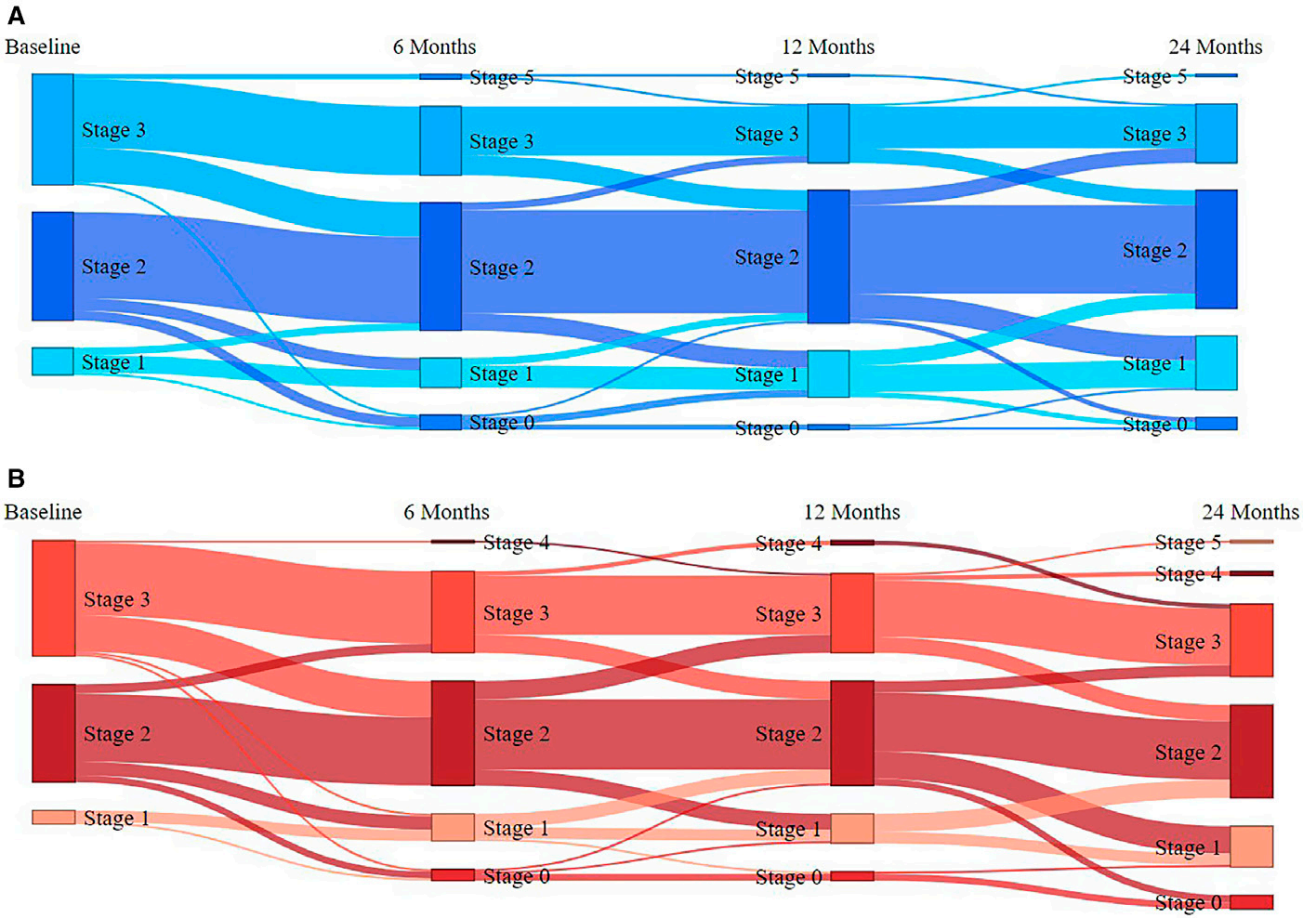
Dynamics of lymphedema changes in patients over the 24-month course of study. (**A**) Doxycycline treatment arm; (**B**) placebo treatment arm.

Few participants in either the treatment or placebo groups experienced progression (worsening) of LE stage, but a substantial number of participants experienced reduction (improvement) in stage of LE over the course of the study (Table 3). This improvement was seen across both treatment and placebo groups, and these groups were not statistically different from each other (*P* = 0.7675). This overall improvement in study participant grade can be seen graphically in a Sankey diagram, which visualizes the stages and stage changes of participants over time for both the doxycycline (Figure 4A) and placebo (Figure 4B) study arms.

**Figure 5.**
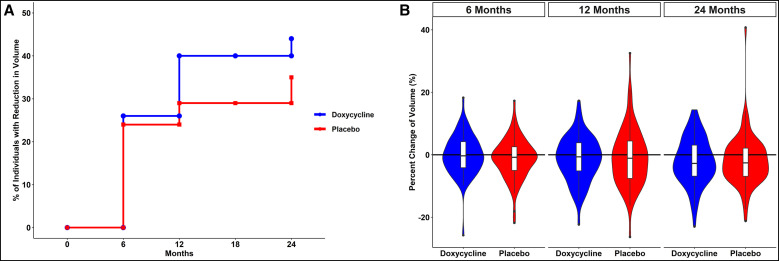
Reduction in lymphedema (LE) volume over the 24-month period of study. (**A**) Percentage of patients receiving placebo (blue) or doxycycline (red) showing reduction in LE volume. (**B**) Violin plots of percent changes in LE volume for both placebo (red) and doxycycline (blue) groups.

Three different methods were used to define the cause of the LE stage changes (see Materials and Methods). As shown in Table 2, at baseline there were no differences in volume measured by LymphaTech between the doxycycline and placebo groups, nor were there differences in skin thickness or circumferential measurements at four defined areas of the affected leg. Although the LE volume was reduced in the overall study population at 24 months by a mean of 48.7 mL (95% CI: –79.63, –17.78), this reduction did not differ significantly between the two study groups (*P* = 0.451). Similarly, there was no difference in proportion of participants showing a LE volume reduction between the doxycycline (43/98; 43.9%) and placebo (34/97; 35%) treatment arms at month 24 (*P* = 0.242) or at the intervening time points (months 6 and 12) (Figure 5). Figure 5A shows the percentage of patients showing reduction in LE volume in those receiving placebo (blue) and those receiving doxycycline (red). Figure 5B shows violin plots of percent changes of volume in LE for both placebo (red) and doxycycline (blue). Furthermore, the reductions in limb volume were similar in the two groups (median reduction, 78 mL in the placebo arm and 73 mL in the doxycycline arm) at 24 months (data not shown), and the volume reductions at 6, 12, and 24 months were also similar between the two treatment arms.

**Figure 6.**
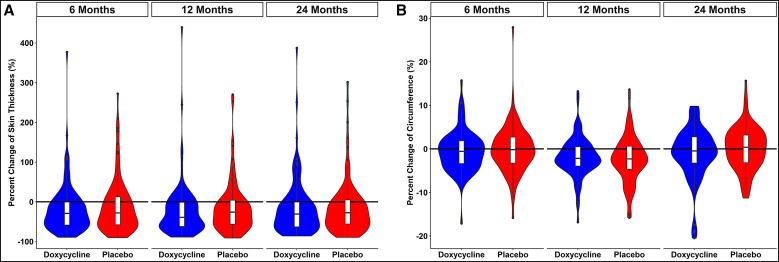
Changes in skin thickness (**A**) and leg circumference (**B**) over 24 months.

Changes in the other measures of LE improvement assessed over time are summarized in Figure 6. Neither the ultrasound-based measurements of skin thickness (Figure 6A) nor circumferential measurements (Figure 6B) of the limbs showed differences between the treatment arms.

**Figure 7.**
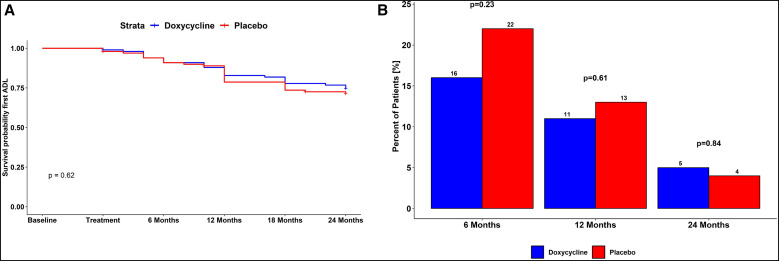
Acute adenolymphangitis (ADLA) attacks over the study period. (**A**) Reduction in number of ADLA attacks over study period. (**B**) Number of ADLA attacks experienced during each time period.

Another way to assess the impact of treatment on LE and/or lymphatic function is to examine the reduction in the number of ADLA attacks over time (Figure 7A). Over the duration of the study, ADLA attacks occurred at roughly the same rate in both groups. The number of ADLA attacks experienced during each of the time periods (0–6 months, 6–12 months, and 12–24 months) gradually decreased (Figure 7B). Of the patients who received doxycycline, only 5% (5/100) reported ADLA attacks occurring between 12 and 24 months (Figure 7B), which did not differ significantly from those in the placebo arm (4/100; *P* = 0.84).

Disability is a major consequence of filarial LE. Over the 24-month period, there was a significant decrease in the mean WHODAS score for all patients in both treatment arms, reflecting a significant improvement in quality of life. This improvement was seen in patients treated with either doxycycline or placebo (Table 4).

**Table 4:** WHODAS score change for each study arm over the 24-month study period

Treatment Arm	Parameter	WHODAS Score at Indicated Follow-up Period		
		Baseline	Month 12	Month 24
Doxycycline	*N*	97	87	92
	Mean ± SEM	20.6 ± 1.3	8.3 ± 0.86	7.8 ± 1.3
	95% CI	18.1–23.1	6.5–10.0	5.3–10.3
Placebo	*N*	97	86	88
	Mean ± SEM	20.21 ± 1.1	10.6 ± 1.1	6.9 ± 1.0
	95% CI	18.0–22.5	8.4–12.8	4.9–8.9

*N* = number; SEM = standard error of the mean; WHODAS = WHO Disability Assessment Schedule.

### Foot care and hygiene.

**Figure 8.**
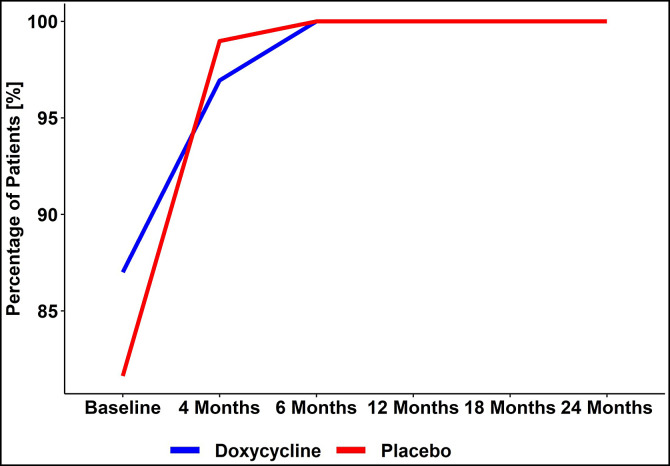
Adherence to hygiene measures over the 24 months of the study.

Attentiveness to hygiene of the affected limbs increased steadily throughout the study (Figure 8). By the end of the study period, patients’ adherence to the hygiene protocol was 100% at each visit. To achieve this, phone calls were made to remind patients about the importance of hygiene between and during study visits. The patients were advised to visit the closest community health center as soon as they observed any abnormal sign or symptom whether related or not to the LE. The local health workers were trained on the use of authorized medications and asked to record any medication provided to the study participants.

### Treatment safety.

In total, 106 separate adverse events were reported. Most of the events observed during the treatment phase were mild and of comparable frequency in the two treatment arms (Table 5). Vomiting after dosing was seen only in patients receiving doxycycline. This is a recognized adverse event that is usually avoided by taking the medication immediately after eating. Many patients had documented fungal infections (intertrigo) that probably resulted from the interaction between LE, a dry dusty environment, and lack of suitable footwear (Table 5). During the follow-up period, a 60-year-old female patient died of a presumed acute cardiovascular event at about 23 months postenrollment.

**Table 5:** Frequency distribution of the most common adverse events observed during treatment in the two treatment arms

Adverse Event	Most Frequently Reported Adverse Events			*P*-Value
	Doxycycline Treatment Arm, *n* (%)	Placebo Treatment Arm, *n* (%)	Total, *N* (%)	
Mycosis/Fungal Infection	13 (39)	20 (61)	33 (100)	0.096
Headache	12 (52)	11 (48)	23 (100)	0.99
Pain in Leg	6 (55)	5 (45)	11 (100)	0.99
Vomiting	**7 (100)**	**0 (0)**	**7 (**100**)**	**0.013**
Chill/Fever	4 (57)	3 (43)	7 (100)	0.99
Leg Wound/Ulcer	4 (57)	3 (43)	7 (100)	0.99
Dyspepsia	3 (60)	2 (40)	5 (100)	0.99
Dizziness/Vertigo	3 (75)	1 (25)	4 (100)	0.619
Nausea	3 (75)	1 (25)	4 (100)	0.619

*n* = number; *N* = total population number. Bold indicates a statistically significant result.

## DISCUSSION

This study was conducted in rural areas of Mali where the patients were widely dispersed in small village communities. Each of the study sites was several hours of travel from the capital (Bamako) and the study laboratories, which posed logistical issues, especially in transporting samples. Facilities at the study sites were minimal before the study, and some infrastructure had to be put in place. By having trained personnel at the study sites on a regular basis, patients remained in contact with the study team throughout, receiving training and support, even when COVID-19 restrictions limited study staff mobility.

### Impact of doxycycline treatment.

By 24 months, the study population showed a significant decrease in affected leg volume from baseline, but there was no significant difference between the doxycycline and placebo groups. Similarly, by 24 months, 38.9% of participants had seen improvement in their LE stage (again with no difference between treatment and placebo groups). These results differ from the study by Mand et al.,^14^ which found a significant decrease in skin thickness in patients treated with doxycycline compared with that of patients treated with either amoxicillin or a placebo after 24 months; this study also showed that doxycycline resulted in an improvement in LE stage compared with those given placebo (37% versus 6%, respectively).

The study by Mand et al. was conducted in 2009, when LF transmission was still ongoing in Ghana, whereas the current study was conducted nearly a decade later, when transmission in Mali had ceased. Doxycycline may have a greater impact when an active filarial infection is ongoing due to not only its impact on *Wolbachia* and/or larval stages of *Onchocerca volvulus* but also on ongoing infection-associated inflammatory processes.^16^ The other important difference between the two studies is the level of hygiene training received. In the earlier study (Mand et al.^14^), some training was given, but this was not supported or sustained, in contrast to the current study, where detailed training and support were given regularly throughout the study.^14^

Although doxycycline combined with enhanced limb hygiene did not appear to provide results superior to those from hygiene alone, the LE volume reduction from baseline to month 24 was clinically perceptible even though not statistically significant either between treatment groups or within each of the two groups. Such findings suggest a continued benefit of hygiene treatment, and it will be interesting to assess the LE volume at month 36 or beyond to have more insight into the longer-term evolution of this reduction in both treatment groups.

Our data showed a reduction in frequency of ADLA attacks in both doxycycline-treated and placebo-treated patients. The two treatment groups were statistically comparable in this regard (*P* = 0.99). At the 6-month study visit, when one might expect an antibiotic effect to be maximal, 16% (16/100) of patients in the doxycycline treatment group had reported ADLA attacks, compared with 23% (22/97) patients in the placebo treatment group. In the entire year prior to the 24-month visit, only 5% (5/100) of doxycycline-treated patients and 4% (4/97) of placebo-treated patients reported ADLA attacks. The progressive reduction in the frequency of ADLA attacks in both study arms could be attributed to the training in limb care and hygiene provided to all patients and their adherence to the training regimens during the study. Indeed, it is well appreciated that limb hygiene prevents secondary bacterial infections responsible for the occurrence of ADLA attacks.^11^ A study conducted in Ethiopia by Douglass et al.^26^ also showed a reduction in frequency of ADLA attacks when limb hygiene was regularly carried out. In Haiti, Addiss et al.^27^ reported a rapid reduction in the incidence of ADLA attacks when hygiene and skin care were emphasized. Several studies have indicated the importance of ADLA attacks in the progression of LE leading to poor quality of life in affected individuals.^14^^,^^16^ Thus, reducing the frequency of ADLA attacks is undoubtedly one of the most important objectives in the management of LE, especially in poor, low-income, remote rural areas of countries such as Mali.

Antibiotic use for conditions not associated with LE was very limited in this study, and therefore there was insufficient opportunity to investigate any potential confounding effects of nonstudy antibiotics on the frequency or severity of ADLA.

### Limb hygiene adherence in rural settings.

Self-care for LE was well accepted because these patients had already tried several treatments (conventional and traditional) that had not been effective for their LE. Their enthusiasm increased especially because they were being trained and supported regularly. The level of compliance could also be due to the relatively older age of our patients (mean age, 52 ± 1.06 years). In Mali, in 2018, during a study on the epidemiological and clinical aspects of LE, Gassama et al.^4^^,^^28^ in urban areas and Dolo et al.^18^ in rural settings also reported that patients are now relatively few in number. This is a sign of successful LF transmission interruption, leaving young people free of LE in Mali, where MDA targeting of LF ceased in 2019 in all implementation units.^9^

In remote rural areas, patient adherence to hygiene-focused self-care needs to be promoted, given the scarcity of health care workers and the finances available in this setting. The low number of LE patients in each individual village should not inhibit provision of support for patients who, despite low literacy levels, have shown excellent compliance when minimal support was made available.^29^ The availability of low-cost care and the repeated training offered by the study team together played pivotal roles in the sustained use of hygiene practices, especially the cleaning of affected limbs.

### Treatment safety.

Most adverse events observed during the treatment period were of mild or moderate intensity and had a comparable frequency between the two treatment groups. The most frequently observed adverse events were cases of intertrigo (31%; 33/106) and headache (22%; 23/106). Vomiting, with seven occurrences in the doxycycline arm only, was significantly more common in that group (*P* = 0.013). These adverse event frequencies were consistent with known doxycycline side effects and with those found by Mand et al.,^14^ who reported no serious adverse events during treatment. The one death occurring during follow-up in the present study was not considered to be treatment related.

## CONCLUSION

Since the effect of doxycycline added to the personal hygiene regimen reported in our study was not superior to that of the use of hygiene alone, patients should be encouraged to use limb hygiene as the main approach to LE management. Local hygiene practices need to be standardized, promoted, and supported in rural settings to increase patient awareness and adherence. The improvement of patients with both reduction in leg volume and a reduced frequency of ADLA deserves extension and assessment beyond the 2 years of this study to determine whether the improvement can be maintained in the absence of continued support for hygiene measures. Indeed, follow-up studies of the factors that impact adherence to standardized hygiene practices for those with LE are planned, and their outcome could significantly influence global policy and practice.

## Supplemental Materials

10.4269/ajtmh.23-0908 Supplemental Materials
